# 4-(3-Nitro­phen­yl)-1-(2-oxoindolin-3-yl­idene)thio­semicarbazide

**DOI:** 10.1107/S1600536810017964

**Published:** 2010-05-22

**Authors:** Humayun Pervez, Mohammad S. Iqbal, Naveeda Saira, Muhammad Yaqub, M. Nawaz Tahir

**Affiliations:** a Department of Chemistry, Bahauddin Zakariya University, Multan 60800, Pakistan; b Department of Chemistry, Government College University, Lahore, Pakistan; c Department of Physics, University of Sargodh, Sargodha, Pakistan

## Abstract

In the title compound, C_15_H_11_N_5_O_3_S, intra­molecular N—H⋯N hydrogen bonding forms an *S*(5) ring motif, whereas N—H⋯O and C—H⋯S inter­actions type complete *S*(6) ring motifs. The 2-oxoindoline and 3-methoxy­phenyl rings are almost planar, with r.m.s. deviations of 0.0178 and 0.0149 Å, respectively, and form a dihedral angle of 33.59 (3)°. In the crystal, mol­ecules are inter­linked through the nitro groups in an end-to-end fashion *via* N—H⋯O and C—H⋯O inter­actions.

## Related literature

For the preparation and structures of biologically important *N* ^4^-aryl-substituted isatin-3-thio­semicarbazones, see: Pervez *et al.* (2007 ▶). For related structures, see: (Pervez *et al.* 2010*a* ▶,*b* ▶). For graph-set notation, see: Bernstein *et al.* (1995 ▶).
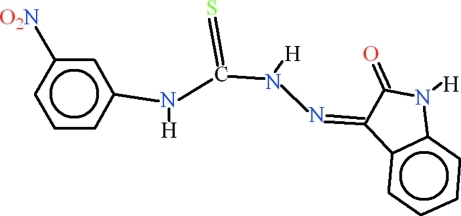


## Experimental

### 

#### Crystal data

C_15_H_11_N_5_O_3_S
*M* *_r_* = 341.35
Orthorhombic, 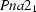

*a* = 18.5545 (10) Å
*b* = 15.3852 (8) Å
*c* = 5.3367 (4) Å
*V* = 1523.44 (16) Å^3^
*Z* = 4
Mo *K*α radiation
μ = 0.24 mm^−1^
*T* = 296 K
0.24 × 0.16 × 0.14 mm

#### Data collection

Bruker Kappa APEXII CCD diffractometer
Absorption correction: multi-scan (*SADABS*; Bruker, 2005 ▶) *T*_min_ = 0.957, *T*_max_ = 0.966
7285 measured reflections
2751 independent reflections
1957 reflections with *I* > 2σ(*I*)
*R*_int_ = 0.036

#### Refinement

*R*[*F* ^2^ > 2σ(*F* ^2^)] = 0.036
*wR*(*F* ^2^) = 0.096
*S* = 1.00
2751 reflections
217 parameters
1 restraint
H-atom parameters constrained
Δρ_max_ = 0.16 e Å^−3^
Δρ_min_ = −0.27 e Å^−3^
Absolute structure: Flack (1983 ▶), 992 Friedel pairs
Flack parameter: −0.05 (11)

### 

Data collection: *APEX2* (Bruker, 2007 ▶); cell refinement: *SAINT* (Bruker, 2007 ▶); data reduction: *SAINT*; program(s) used to solve structure: *SHELXS97* (Sheldrick, 2008 ▶); program(s) used to refine structure: *SHELXL97* (Sheldrick, 2008 ▶); molecular graphics: *ORTEP-3 for Windows* (Farrugia, 1997 ▶) and *PLATON* (Spek, 2009 ▶); software used to prepare material for publication: *WinGX* (Farrugia, 1999 ▶) and *PLATON*.

## Supplementary Material

Crystal structure: contains datablocks global, I. DOI: 10.1107/S1600536810017964/bq2210sup1.cif

Structure factors: contains datablocks I. DOI: 10.1107/S1600536810017964/bq2210Isup2.hkl

Additional supplementary materials:  crystallographic information; 3D view; checkCIF report

## Figures and Tables

**Table 1:** Hydrogen-bond geometry (Å, °)

*D*—H⋯*A*	*D*—H	H⋯*A*	*D*⋯*A*	*D*—H⋯*A*
N1—H1⋯O3^i^	0.86	2.21	3.058 (3)	170
N3—H3⋯O1	0.86	2.13	2.797 (3)	134
N4—H4*A*⋯N2	0.86	2.12	2.580 (3)	113
C7—H7⋯O2^i^	0.93	2.55	3.358 (4)	145
C11—H11⋯S1	0.93	2.56	3.204 (3)	127

Symmetry code: (i) 
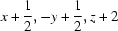
.

**Fig. 1.**
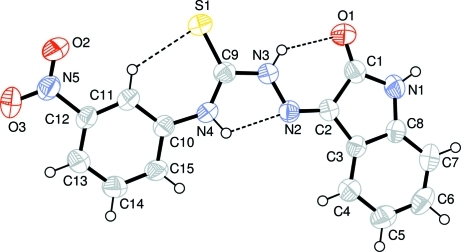
View of the title compound with the atom numbering scheme. The thermal ellipsoids are drawn at the 50% probability level. The dotted lines indicate the intra-molecular H-bondings.

**Fig. 2.**
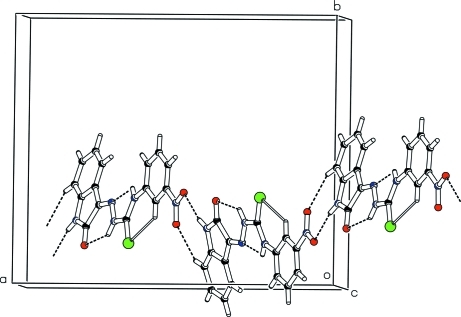
The partial packing (PLATON; Spek, 2009) which shows that molecules form dimers which are interlinked.

## supplementary crystallographic information

### Comment

As a part of our work on the synthesis of certain biologically important isatin derivatives (Pervez *et al.*, 2007), we report herein the structure and synthesis of the title compound (I, Fig. 1).

The crystal structure of (II) i.e. 4-(2-fluorophenyl)-1-(2-oxoindolin-3-ylidene)thiosemicarbazide (Pervez *et al.*, 2010b) and (III) i.e. 4-(3-methoxyphenyl)-1-(2-oxoindolin-3-ylidene)thiosemicarbazide (Pervez *et al.*, 2010a) have been reported. The title compound (I) differs from (II) due to the attachment of nitro group at position-3 instead of fluoro at position-2 of the phenyl ring substituted at N^4^ of the thiosemicarbazone moiety. Similarly (I) differs from (III) due to the presence of nitro instead of methoxy function at position-3 of the phenyl ring. In (I) the 2-oxoindolin A (C1–C8/N1/O1), thiosemicarbazide B (N2/N3/C9/S1/N4) and the 3-methoxyphenyl C (C10—C16/O2) are planar with r. m. s. deviations of 0.0178, 0.0244 and 0.0149 Å, respectively. The dihedral angle between A/B, A/C and B/C is 8.71 (5)°, 33.59 (3)° and 39.32 (3)°, respectively. Due to intramolecular H-bondings (Table 1, Fig. 1), one S(5) and two S(6) (Bernstein *et al.*, 1995) ring motifs are formed. The molecules are interlinked through nitro groups (Fig. 2) in end to end fashion due to N—H···O and C–H···O interactions completing R_2_^2^(8) ring motifs. The N=O···π and C=S···π interaction play role in stabilizing the molecules.

### Experimental

To a hot solution of isatin (0.74 g, 5.0 mmol) in ethanol (10 ml) containing a few drops of glacial acetic acid was added 4-(3-nitrophenyl)thiosemicarbazide (1.06 g, 5.0 mmol) dissolved in ethanol (10 ml) under stirring. The reaction mixture was then heated under reflux for 2 h. The orange crystalline solid formed during heating was collected by suction filtration. Thorough washing with hot ethanol followed by ether provided the desired compound (I) in pure form (1.08 g, 63%), m.p. 539 K. The single crystals of (I) were grown in ethyl acetate by slow evaporation at room temperature.

### Refinement

The H-atoms were positioned geometrically (N–H = 0.86 Å, C–H = 0.93 Å) and refined as riding with *U*_iso_(H) = x*U*_eq_(C, N), where x = 1.2 for all H-atoms.

### Crystal data

**Table:**

C_15_H_11_N_5_O_3_S	*F*(000) = 704
*M**_r_* = 341.35	*D*_x_ = 1.488 Mg m^−^^3^
Orthorhombic, *P**n**a*2_1_	Mo *K*α radiation, λ = 0.71073 Å
Hall symbol: P 2c -2n	Cell parameters from 2751 reflections
*a* = 18.5545 (10) Å	θ = 2.6–26.5°
*b* = 15.3852 (8) Å	µ = 0.24 mm^−^^1^
*c* = 5.3367 (4) Å	*T* = 296 K
*V* = 1523.44 (16) Å^3^	Prism, light yellow
*Z* = 4	0.24 × 0.16 × 0.14 mm

### Data collection

**Table:**

Bruker Kappa APEXII CCD diffractometer	2751 independent reflections
Radiation source: fine-focus sealed tube	1957 reflections with *I* > 2σ(*I*)
graphite	*R*_int_ = 0.036
Detector resolution: 7.80 pixels mm^-1^	θ_max_ = 26.5°, θ_min_ = 2.6°
ω scans	*h* = −23→23
Absorption correction: multi-scan (*SADABS*; Bruker, 2005)	*k* = −19→18
*T*_min_ = 0.957, *T*_max_ = 0.966	*l* = −6→5
7285 measured reflections	

### Refinement

**Table:**

Refinement on *F*^2^	Secondary atom site location: difference Fourier map
Least-squares matrix: full	Hydrogen site location: inferred from neighbouring sites
*R*[*F*^2^ > 2σ(*F*^2^)] = 0.036	H-atom parameters constrained
*wR*(*F*^2^) = 0.096	*w* = 1/[σ^2^(*F*_o_^2^) + (0.0476*P*)^2^] where *P* = (*F*_o_^2^ + 2*F*_c_^2^)/3
*S* = 1.00	(Δ/σ)_max_ < 0.001
2751 reflections	Δρ_max_ = 0.16 e Å^−^^3^
217 parameters	Δρ_min_ = −0.27 e Å^−^^3^
1 restraint	Absolute structure: Flack (1983), 992 Friedel pairs
Primary atom site location: structure-invariant direct methods	Flack parameter: −0.05 (11)

### Special details

**Table:**

Geometry. All esds (except the esd in the dihedral angle between two l.s. planes) are estimated using the full covariance matrix. The cell esds are taken into account individually in the estimation of esds in distances, angles and torsion angles; correlations between esds in cell parameters are only used when they are defined by crystal symmetry. An approximate (isotropic) treatment of cell esds is used for estimating esds involving l.s. planes.
Refinement. Refinement of *F*^2^ against ALL reflections. The weighted *R*-factor *wR* and goodness of fit *S* are based on *F*^2^, conventional *R*-factors *R* are based on *F*, with *F* set to zero for negative *F*^2^. The threshold expression of *F*^2^ > σ(*F*^2^) is used only for calculating *R*-factors(gt) *etc*. and is not relevant to the choice of reflections for refinement. *R*-factors based on *F*^2^ are statistically about twice as large as those based on *F*, and *R*- factors based on ALL data will be even larger.

### Fractional atomic coordinates and isotropic or equivalent isotropic displacement parameters (Å^2^)

**Table:**

	*x*	*y*	*z*	*U*_iso_*/*U*_eq_	
S1	0.22195 (4)	0.31899 (4)	−0.2161 (2)	0.0691 (3)	
O1	0.38865 (11)	0.31688 (13)	0.4271 (5)	0.0693 (6)	
O2	0.04767 (14)	0.25693 (14)	−0.7833 (6)	0.0987 (11)	
O3	0.01628 (11)	0.14340 (14)	−0.9884 (5)	0.0691 (6)	
N1	0.42883 (11)	0.21951 (15)	0.7293 (5)	0.0584 (7)	
H1	0.4577	0.2529	0.8109	0.070*	
N2	0.29869 (10)	0.15983 (12)	0.2774 (5)	0.0462 (5)	
N3	0.28351 (11)	0.23206 (14)	0.1419 (5)	0.0511 (6)	
H3	0.3036	0.2806	0.1808	0.061*	
N4	0.21013 (11)	0.14865 (14)	−0.0938 (5)	0.0485 (6)	
H4A	0.2223	0.1111	0.0179	0.058*	
N5	0.04756 (12)	0.17858 (16)	−0.8135 (5)	0.0573 (7)	
C1	0.38904 (15)	0.24541 (19)	0.5275 (6)	0.0526 (8)	
C2	0.34565 (13)	0.16630 (16)	0.4526 (6)	0.0443 (6)	
C3	0.36668 (12)	0.09693 (16)	0.6207 (6)	0.0440 (6)	
C4	0.34648 (14)	0.01067 (17)	0.6375 (6)	0.0529 (7)	
H4	0.3137	−0.0131	0.5247	0.063*	
C5	0.37617 (16)	−0.03939 (19)	0.8262 (7)	0.0627 (9)	
H5	0.3632	−0.0975	0.8415	0.075*	
C6	0.42479 (16)	−0.0037 (2)	0.9915 (7)	0.0666 (9)	
H6	0.4435	−0.0385	1.1182	0.080*	
C7	0.44684 (14)	0.0822 (2)	0.9761 (6)	0.0596 (8)	
H7	0.4802	0.1054	1.0876	0.072*	
C8	0.41680 (12)	0.13180 (17)	0.7871 (7)	0.0483 (6)	
C9	0.23686 (13)	0.22885 (16)	−0.0549 (6)	0.0463 (7)	
C10	0.16580 (12)	0.11539 (16)	−0.2849 (5)	0.0448 (7)	
C11	0.12759 (13)	0.16537 (16)	−0.4543 (6)	0.0475 (7)	
H11	0.1294	0.2257	−0.4476	0.057*	
C12	0.08673 (13)	0.12370 (18)	−0.6333 (6)	0.0467 (7)	
C13	0.08131 (14)	0.03494 (18)	−0.6548 (7)	0.0594 (9)	
H13	0.0538	0.0090	−0.7799	0.071*	
C14	0.11867 (17)	−0.01353 (19)	−0.4821 (8)	0.0669 (10)	
H14	0.1158	−0.0738	−0.4884	0.080*	
C15	0.16025 (13)	0.02508 (17)	−0.3002 (7)	0.0564 (8)	
H15	0.1850	−0.0094	−0.1859	0.068*	

### Atomic displacement parameters (Å^2^)

**Table:**

	*U*^11^	*U*^22^	*U*^33^	*U*^12^	*U*^13^	*U*^23^
S1	0.0952 (5)	0.0440 (4)	0.0682 (6)	0.0028 (4)	−0.0217 (6)	0.0061 (4)
O1	0.0843 (14)	0.0538 (12)	0.0697 (17)	−0.0173 (10)	−0.0095 (13)	0.0108 (11)
O2	0.1169 (18)	0.0549 (14)	0.124 (3)	−0.0056 (12)	−0.060 (2)	0.0259 (15)
O3	0.0610 (12)	0.0922 (16)	0.0540 (15)	0.0020 (11)	−0.0169 (12)	0.0025 (12)
N1	0.0572 (13)	0.0627 (15)	0.055 (2)	−0.0116 (10)	−0.0129 (13)	−0.0018 (13)
N2	0.0479 (11)	0.0467 (13)	0.0439 (15)	0.0030 (8)	−0.0014 (13)	−0.0011 (13)
N3	0.0608 (14)	0.0423 (13)	0.0501 (17)	−0.0004 (9)	−0.0107 (13)	0.0009 (11)
N4	0.0552 (12)	0.0424 (12)	0.0478 (16)	0.0017 (10)	−0.0072 (11)	0.0080 (10)
N5	0.0487 (12)	0.0632 (17)	0.060 (2)	−0.0013 (11)	−0.0065 (13)	0.0151 (14)
C1	0.0522 (15)	0.0573 (18)	0.048 (2)	−0.0067 (13)	0.0022 (15)	−0.0028 (15)
C2	0.0422 (13)	0.0482 (16)	0.0425 (18)	−0.0008 (10)	−0.0003 (13)	−0.0025 (12)
C3	0.0435 (13)	0.0491 (16)	0.0396 (18)	0.0049 (11)	0.0015 (13)	−0.0005 (13)
C4	0.0522 (15)	0.0536 (18)	0.053 (2)	0.0024 (12)	0.0011 (14)	0.0009 (14)
C5	0.0688 (17)	0.0548 (17)	0.064 (3)	0.0099 (13)	0.0049 (19)	0.0084 (16)
C6	0.0682 (19)	0.079 (2)	0.053 (2)	0.0252 (16)	0.0012 (17)	0.0140 (17)
C7	0.0555 (16)	0.080 (2)	0.043 (2)	0.0080 (15)	−0.0047 (15)	−0.0012 (15)
C8	0.0467 (12)	0.0569 (17)	0.0414 (18)	0.0025 (11)	0.0002 (16)	−0.0004 (15)
C9	0.0505 (13)	0.0425 (15)	0.0458 (19)	0.0059 (12)	0.0039 (15)	−0.0024 (13)
C10	0.0415 (12)	0.0475 (16)	0.045 (2)	0.0023 (10)	0.0007 (12)	0.0028 (12)
C11	0.0471 (14)	0.0470 (15)	0.0482 (19)	0.0040 (11)	−0.0006 (13)	0.0067 (13)
C12	0.0391 (11)	0.0543 (16)	0.047 (2)	0.0007 (12)	−0.0016 (12)	0.0111 (13)
C13	0.0543 (15)	0.0562 (18)	0.068 (3)	−0.0087 (13)	−0.0116 (17)	0.0010 (15)
C14	0.0692 (18)	0.0454 (16)	0.086 (3)	−0.0022 (14)	−0.0198 (19)	0.0051 (17)
C15	0.0495 (14)	0.0459 (16)	0.074 (2)	−0.0006 (12)	−0.0134 (16)	0.0120 (14)

### Geometric parameters (Å, °)

**Table:**

S1—C9	1.655 (3)	C4—C5	1.382 (4)
O1—C1	1.223 (3)	C4—H4	0.9300
O2—N5	1.216 (3)	C5—C6	1.376 (5)
O3—N5	1.225 (3)	C5—H5	0.9300
N1—C1	1.365 (4)	C6—C7	1.386 (4)
N1—C8	1.402 (3)	C6—H6	0.9300
N1—H1	0.8600	C7—C8	1.382 (4)
N2—C2	1.282 (4)	C7—H7	0.9300
N2—N3	1.355 (3)	C10—C11	1.382 (4)
N3—C9	1.362 (4)	C10—C15	1.396 (3)
N3—H3	0.8600	C11—C12	1.378 (4)
N4—C9	1.346 (3)	C11—H11	0.9300
N4—C10	1.407 (3)	C12—C13	1.374 (4)
N4—H4A	0.8600	C13—C14	1.373 (4)
N5—C12	1.472 (4)	C13—H13	0.9300
C1—C2	1.513 (4)	C14—C15	1.375 (4)
C2—C3	1.448 (4)	C14—H14	0.9300
C3—C4	1.382 (3)	C15—H15	0.9300
C3—C8	1.393 (4)		
			
C1—N1—C8	111.6 (2)	C5—C6—H6	118.8
C1—N1—H1	124.2	C7—C6—H6	118.8
C8—N1—H1	124.2	C8—C7—C6	116.8 (3)
C2—N2—N3	117.8 (2)	C8—C7—H7	121.6
N2—N3—C9	120.9 (2)	C6—C7—H7	121.6
N2—N3—H3	119.5	C7—C8—C3	121.4 (2)
C9—N3—H3	119.5	C7—C8—N1	128.9 (3)
C9—N4—C10	131.3 (2)	C3—C8—N1	109.7 (3)
C9—N4—H4A	114.3	N4—C9—N3	112.7 (2)
C10—N4—H4A	114.3	N4—C9—S1	128.8 (2)
O2—N5—O3	122.7 (3)	N3—C9—S1	118.5 (2)
O2—N5—C12	118.7 (3)	C11—C10—C15	118.5 (3)
O3—N5—C12	118.6 (2)	C11—C10—N4	124.9 (2)
O1—C1—N1	127.7 (3)	C15—C10—N4	116.6 (2)
O1—C1—C2	127.2 (3)	C12—C11—C10	118.5 (2)
N1—C1—C2	105.2 (2)	C12—C11—H11	120.8
N2—C2—C3	125.3 (2)	C10—C11—H11	120.8
N2—C2—C1	128.1 (2)	C13—C12—C11	124.1 (3)
C3—C2—C1	106.6 (2)	C13—C12—N5	118.6 (3)
C4—C3—C8	120.6 (3)	C11—C12—N5	117.3 (2)
C4—C3—C2	132.5 (3)	C14—C13—C12	116.5 (3)
C8—C3—C2	106.9 (2)	C14—C13—H13	121.7
C3—C4—C5	118.3 (3)	C12—C13—H13	121.7
C3—C4—H4	120.8	C13—C14—C15	121.5 (3)
C5—C4—H4	120.8	C13—C14—H14	119.2
C6—C5—C4	120.4 (3)	C15—C14—H14	119.2
C6—C5—H5	119.8	C14—C15—C10	120.8 (3)
C4—C5—H5	119.8	C14—C15—H15	119.6
C5—C6—C7	122.4 (3)	C10—C15—H15	119.6

### Hydrogen-bond geometry (Å, °)

**Table:**

*D*—H···*A*	*D*—H	H···*A*	*D*···*A*	*D*—H···*A*
N1—H1···O3^i^	0.86	2.21	3.058 (3)	170
N3—H3···O1	0.86	2.13	2.797 (3)	134
N4—H4A···N2	0.86	2.12	2.580 (3)	113
C7—H7···O2^i^	0.93	2.55	3.358 (4)	145
C11—H11···S1	0.93	2.56	3.204 (3)	127

Symmetry codes: (i) *x*+1/2, −*y*+1/2, *z*+2.
